# Characteristics associated with pediatric growth measurement collection in electronic medical records: a retrospective observational study

**DOI:** 10.1186/s12875-020-01259-x

**Published:** 2020-09-15

**Authors:** Leanne Kosowan, John Page, Jennifer Protudjer, Tyler Williamson, John Queenan, Alexander Singer

**Affiliations:** 1 Department of Family Medicine, Rady Faculty of Health Sciences, University of Manitoba, Winnipeg, Manitoba Canada; 2 The Children’s Hospital of Winnipeg, Winnipeg, Manitoba Canada; 3 Centre for Healthcare Innovation, University of Manitoba, Winnipeg, Manitoba Canada; 4 Departments of Biostatistics & Community Health Sciences, University of Calgary, Calgary, Alberta Canada; 5 Centre for Studies in Primary Care, Department of Family Medicine, Queen’s University, Kingston, Ontario Canada

**Keywords:** Primary health care, Electronic health records, Growth and development, Child development

## Abstract

**Background:**

Complete growth measurements are an essential part of pediatric care providing a proxy for a child’s overall health. This study describes the frequency of well-child visits, documented growth measurements, and clinic and provider factors associated with measurement.

**Methods:**

Retrospective cross-sectional study utilizing electronic medical records (EMRs) from primary care clinics between 2015 and 2017 in Manitoba, Canada. This study assessed the presence of recorded height, weight and head circumference among children (0–24 months) who visited one of 212 providers participating in the Manitoba Primary Care Research Network. Descriptive and multivariable logistic regression analyses assessed clinic, provider, and patient factors associated with children having complete growth measurements.

**Results:**

Our sample included 4369 children. The most frequent growth measure recorded was weight (79.2% *n* = 3460) followed by height (70.8% *n* = 3093) and head circumference (51.4% *n* = 2246). 67.5% of children (*n* = 2947) had at least one complete growth measurement recorded (i.e. weight, height and head circumference) and 13.7% (*n* = 599) had complete growth measurements at all well-child intervals attended. Pediatricians had 2.7 higher odds of documenting complete growth measures within well-child intervals compared to family physicians (95% CI 1.8–3.8). Additionally, urban located clinics (OR 1.7, 95% CI 1.2–2.5), Canadian trained providers (OR 2.3, 95% CI 1.4–3.7), small practice size (OR 1.6, 95% CI 1.2–2.2) and salaried providers (OR 3.4, 95% CI 2.2–5.2) had higher odds of documented growth measures.

**Conclusions:**

Growth measurements are recorded in EMRs but documentation is variable based on clinic and provider factors. Pediatric growth measures at primary care appointments can improve primary prevention and surveillance of child health outcomes.

## Background

Growth measurements serve as a proxy for a child’s health. Routine growth monitoring is promoted in numerous professional guidelines and widely accepted as a standard of well-child care [1, 2]. It is recommend that providers of well-child care record complete growth measurements of naked weight, recumbent height (i.e. length), and head circumference for all children 0 to 24 months of age [1, 3–9]. Measurement is recommended at regular intervals corresponding to “well child checks” to assess growth velocity (i.e. 2 weeks and 1, 2, 4, 6, 9, 12, 18, and 24 months) [2]. Growth measurement should also occur at acute care visits in children [1, 2, 10]. Deviation from an expected growth trajectory can be the first indication of a potential problem, including an illness or nutritional deficit, and is a critical time for primary prevention strategies [1–3, 11–14]. Nutritional or contextual factors, endocrinopathy or chronic disease may affect growth. Growth deviations are often a result of inadequate nutrition, however it may also signify a disease in an otherwise asymptomatic child [3]. Several conditions present as failure-to-thrive and are screened for using growth measures [14]. The increasing prevalence of obesity among children and adolescents highlights the need to document and monitor child growth [15].

Inconsistent and missing documentation of growth measures in the EMR limits patient-specific primary prevention counselling that could be targeted toward the child and their family, as well as national and provincial surveillance [4]. Strategies aimed at increasing documentation of growth measures in the EMR should be directed towards practices and providers less likely to have documented growth measures [16, 17]. However most of the literature currently available is focused on inpatient populations and the pediatric care setting [14, 15, 18–22]. These settings do not represent the care of children in the community. Documented growth measures extracted from the electronic medical records (EMR) of primary care community-based practices can provide a good proxy for child health surveillance including population obesity risk [11, 15].

## Methods

### Design and setting

This retrospective, cross-sectional study aims to assess attendance at well-child visits among children 0 to 24 months of age, as well as the completeness of growth measurements recorded in the EMR of community-based clinics. This study explores the documentation of growth measures from primary care encounters with 212 primary care providers participating in the Manitoba Primary Care Research Network (MaPCReN) in Manitoba Canada. MaPCReN extracts de-identified information from the EMR of consenting family physicians, nurse practitioners and community pediatricians, and provides semi-annual feedback reports to practices characterizing their patients. The number of clinics and primary care providers participating in MaPCReN continues to increase. Currently, MaPCReN represents approximately 20% of Manitoba’s primary care family physicians and pediatricians [23]. MaPCReN is one of the provincial networks within the Canadian Primary Care Sentential Surveillance Network (CPCSSN). Prior studies have shown that the patient population within CPCSSN is representative of the general population in terms of disease prevalence and prescribing rates when compared to other national data sources [24].

The cohort for this study included 4369 children aged 0–24 months, with at least one visit to a participating MaPCReN provider between June 30, 2015 and June 30, 2017.

### Measures

All encounters to a participating provider were included in this study, independent of the reason for the encounter. The date of the encounter and patient’s birth date were used to place the encounter into an intervals coinciding with the well-child visit recommendations. Eight intervals were considered < 1 month, 1 to 3 months, 3 to 5 months, 5 to 7 months, 7 to 10 months, 10 to 13 months, 13 to 19 months, 19 to 25 months. To be assessed for growth measures (i.e. height, weight, and head circumference) during a well-child interval a child had to have an encounter within that well-child visit interval. Attendance at a well-child appointment is not always within the control of the provider; it is therefore suggested that growth measurements should also be taken at acute care appointments [1, 2, 15]. We did not focus exclusively on the well-child appointments. Instead we assessed for growth measures within the well-child intervals. Therefore, all encounters within the interval was assessed for documented growth measurements. The number of growth measures recorded in the EMR at each encounter was counted. The encounters of each child were separated into the well-child intervals. The encounter with the highest number of growth measures recorded were retained for the analysis. If there was more than one encounter with the same number of measurements recorded in a well-child interval the first visit was retained for analysis. “Complete growth measurements” was defined as documentation of all three measures (i.e. weight, height, and head circumference) at the same appointment. Patients who had complete growth measurements at every well-child visit interval they attended were defined as “fully complete”.

### Covariates

Urban and rural clinic location was determined using the three digit postal code of the clinic. EMR duration and practice size were dichotomized and represented as higher than the mean (6.3 years and 1708 patients, respectively). The providers within our study were hired under one of two funding models; salaried providers were compared to fee-for-service providers who are remunerated based on the billing records submitted to Manitoba Health for payment.

### Statistical analysis

Descriptive statistics were used to explore attendance to well-child visits and growth measurements at the attended visits. We performed a generalized estimate equation (GEE) model with logit function to assess associations between “fully complete” growth measures (yes vs. no) and provider (i.e. provider type (pediatrician vs. family physician), country of graduation (Canadian vs. international graduate), age (continuous), sex (male provider vs. female provider), funding model (salaried vs. fee-for-service), practice size (< 1708 patients vs. ≥1708 patients), practice location (urban vs rural clinic location), length of time using an EMR (< 6.3 years vs. ≥6.3 years)), and patient (i.e. male vs female sex) characteristics. The GEE model considered repetition of provider to control for practice size of the provider within the model. Odds ratios (OR) and 95% confidence intervals (CI) are reported. SAS version 9.4 (SAS Institute, Cary NC) was used for analyses. Ethics approval for this study was obtained from the Health Research Ethics Board at the University of Manitoba. (HS21333(H2017–395)).

## Results

There were 4369 children aged 0 to 24 months whom obtained care from 212 primary care providers. This represents approximately 13% of children in Manitoba. The majority of providers within MaPCReN were family physicians (*n* = 159), followed by nurse practitioners (*n* = 35), and pediatricians (*n* = 18). There were 23 urban clinics and 17 rural clinic locations across Manitoba (Table 1). The average provider age was 43.1 years old (9.7 SD).

**Table 1:** Distribution of Pediatric Patients by Clinic, Provider and Patient Demographics

*N* = 4369		
Variable	N	Proportion
Male patient (vs. female patient)	2314	53.0%
Urban location (vs. rural location)	2315	53.0%
Family Physician	2229	51.0%
Nurse Practitioner	195	4.5%
Pediatrician	1945	44.5%
Female provider (vs. male provider)	2463	56.4%
Canadian Medical Graduate (vs. international medical graduate)	3481	79.7%
Salaried Provider (vs. Fee-for-service provider)	2023	46.3%

Fifty-five percent of the encounters to children aged 0 to 24 months between 2015 and 2017 were billed as a well-child visit. On average children that saw a pediatrician had one additional visit a year to their primary care provider compared to children that saw a family physician or nurse practitioner (7.3 (SD 5.4) vs. 6.3 (SD 3.9), respectively). Pediatricians were twice as likely to bill an encounter for a well-child visit compared to family physicians or nurse practitioners (8.9 (SD 7.6) vs. 4.5 (SD4.0) well-child visits a year). The average number of well-child visits attended per child was 6.5 (SD 6.2) visits over the two-year period. However, there were 327 children (21.2%) that attended an appointment with their physician or pediatrician during this time period (0–24 months of age) but did not have any well-child visits. The well-child interval most attended was 1 to 3 months with 83.5% of patients attending a visit within this interval. Conversely, patients were least likely to attend a visit between 19 and 25 months with 56.8% of the patients attending an appointment at this interval (Table 2). The attendance at each visit interval averaged 67.8% (9.1 SD). In total, 606 (13.9%) patients attended appointments at all eight suggested intervals. Patients who had an encounter at each of the intervals were significantly more likely to have visited a pediatrician (63.0%) compared to a family physician (33.3%) (*p*-value < 0.001).

**Table 2:** Proportion of Patients with an Encounter at each of the Suggested Well-Child Intervals

*N* = 4369	
Well-child Interval	Proportion of Patients
0 - < 1 Month	59.5%
1 - < 3 Months	83.5%
3 - < 5 Months	70.9%
5 - < 7 Months	70.0%
7 - < 10 Months	68.7%
10 - < 13 Months	58.6%
13 - < 19 Months	74.7%
19 - < 25 Months	56.8%

**Fig. 1:**
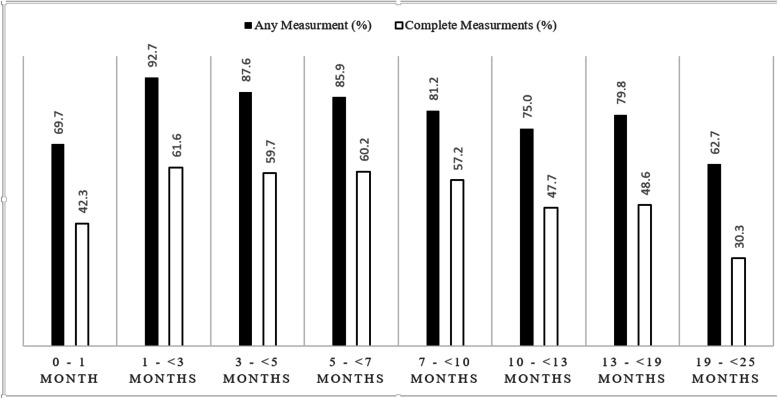
Documented growth measurement (≥1 measurement) and complete measurements of weight, height and head circumference in the electronic medical records of pediatric patients (aged 0-24 months) that attended an appointment with a provider participating in MaPCReN

Growth measures were most likely to be recorded at the 1 to 3 month interval (92.7%) and were least likely to occur at the 19 to 25 month interval (62.7%) (Fig. 1). On average, 79.3% of patients at each interval had a growth measurement recorded in the EMR. Weight was the most frequent growth measure documented (79.2%). Documentation of height occurred on average at 70.8% of the intervals. Documented head circumference was the least likely growth measure to occur within any well-child visit interval (53·8%). On average, complete growth measurements occurred at 51% of assessed encounters. Complete growth measures were least likely to occur at the 19 to 25 month well-child interval (30.3%) and most likely to occur at the 1 to 3 month well-child interval (61.6%) (Fig. 1). In total, 67.5% (*n* = 2947) of the children had at least one complete growth measurement. However, only 13.7% (*n* = 598) had fully complete growth measurements.

There were 598 patients who had fully complete growth measurements recorded at each of the intervals attended. Children with fully complete growth measures had 1.7 times higher odds of visiting an urban clinic (95% CI 1.2–2.5) compared to a rural clinic and 1.6 times higher odds of visiting a smaller than average practice (95% CI 1.2–2.2) compared to a larger than average practice. Children with ‘fully complete’ growth measures had 2.7 times higher odds of having an encounter with a pediatrician compared to a family physician (95% CI 1.8–3.8). Children with a fully complete growth measure had 2.3 times higher odds of visiting a provider trained in Canada compared to a provider trained internationally (95% CI 1.4–3.7), and 3.4 times higher odds of having an encounter with a salaried provider instead of a fee-for-service provider (95% CI 2.2–5.2) (Table 3).

**Table 3:** Multivariable Logit Models with Generalized estimate equation model for ‘Fully complete’ growth measures at 2 years of age

*N* = 4369			
Variable	Odds Ratio	95% CI	***p***-value
Patient Factors			
Male patient (vs. female patients)	0.96	0.8–1.2	0.684
Clinic Factors			
Urban (vs rural)	1.67	1.2–2.5	0.011
EMR duration < 6.3 years (vs ≥6.3 years)	1.63	1.0–2.6	0.041
Provider Factors			
Pediatrician (vs Family physician)	2.65	1.8–3.8	< 0.001
Canadian medical graduate (vs. international medical graduate)	2.25	1.4–3.7	0.001
Salaried provider (vs. Fee-for-service provider)	3.41	2.2–5.2	< 0.001
Provider age (every 1 year increase)	1.01	0.9–1.0	0.256
Male provider (vs. female provider)	1.09	0.8–1.5	0.598
Practice size < 1708 patients (vs. ≥1708 patients)	1.59	1.2–2.2	0.004

## Discussion

Attendance at well-child appointments and documentation of growth measurements are widely recommended for early detection of potential genetic, medical, nutritional, or environmental problems. Delayed or restricted growth suggests the need for corrective interventions and monitoring to ensure full growth potential [1–3, 5–9]. However, despite having a publicly funded health system in Canada, our study found that 21% of children did not attend a well-child appointment between the ages of 0 to 24 months. Pediatricians and family physicians both had patients without any well-child visits. Wolf et al. undertook a qualitative study to identify reasons for missed well-child appointments suggesting barriers to attendance included transportation, difficulty taking time off work, child care, and other social stressors [25].

We found that although visit frequency was similar between patients that saw a family physician and pediatrician, pediatricians were significantly more likely to have billed for a well-child visit, have documented complete growth measures and have children with complete growth measures at all intervals. Similarly, using data from the National Ambulatory Medical Care and National Hospital Ambulatory Medical Care Surveys Burman et al. found that non-pediatricians in the USA were 2.6 times more likely to have undocumented growth measures at pediatric outpatient visits compared to pediatricians [10]. Failure to record growth measures at a well-child appointment represents a missed opportunity to identify early signs of health conditions or introduce primary prevention interventions. Providers who rely on clinical appearance instead of growth measures may not notice early signs of growth deviations [10, 16, 20].

We found that within community-based primary care centers complete growth measurements occur twice as frequently at appointments between months 1 to 3, compared with 19 to 25 months of age. This is consistent with other studies that assessed the EMR of specialty or inpatient centers and found documentation of growth measures in the EMR decreased as children aged [18, 20]. Using data from the National Survey of America’s Families, Yu et al. reported that 79.2% of children had a recorded height and weight measure at 1–3 months, which decreases to only 18% by age 11 years [26]. The decreased documentation of growth measurement during 19 to 25 month timeframe may be related to lack of recommended immunizations at this age [27]. There may be other social factors that play a role in attendance at these visits such as subsequent pregnancy or socioeconomic factors [25, 28]. Inadequate or inaccurate documentation in the EMR can complicate follow-up visits and the ability to assess growth velocity [22].

Similar to other studies, weight was the most commonly recorded growth measure (79.2%), followed by height (70.8%), and finally head circumference (51.4%) [19, 21]. Deviations in height and weight can represent a different health concerns. Endocrinopathy usually affects height more than weight, whereas nutritional problems and systemic disease can affect weight first than height [3]. Weight is required to correctly prescribe numerous pediatric medications, which may explain its increased presence in EMR data [19, 21]. Previous studies have also reported that head circumference is the least common growth measurement, which may be related to it being perceived as cumbersome to measure [26]. Despite this, head circumference is important to record, trend, and interpret as it can indicate a significant genetic or medical condition that requires further evaluation [13, 29].

Even though the rates of complete growth measurements averaged 51%, each well-child visit interval ranged from 30.3 to 61.4%. Only 13.6% of patients had complete growth measurements in each well-child interval they attended. Lipman et al. found that despite guidelines suggesting the interval for growth monitoring, 10% of pediatric practices and 41% of family practices in an inpatient setting report not measuring children at every well-child visit [20]. Additionally, children who are frequently ill or have a chronic condition may not attend well-child visits at all suggested intervals; although these children require care frequently they may not have recorded growth measurements [20]. Interestingly, despite the utility for disease screening, no study has evaluated the effects of growth monitoring on morbidity or mortality, thus there is insufficient evidence to conclude definitively that growth monitoring has a direct health benefit [4, 17].

Children with complete growth measurements at every attended visit interval were more likely to have visited an urban clinic, smaller practice and salaried provider. These providers may be able to take more time with each patient. Jamal et al. also found that clinic factors dictate documentation of growth measures, suggesting that clinic protocols may be an important focus for improvement of captured growth measures [30]. Interestingly we also found that location of medical training affected documentation of growth measures. Providers trained in Canada were more likely to have fully complete growth measure documented in the EMR. Large-scale interventions focused on EMR computer-assisted decision tools for child and adolescents have been shown to increase documented height and weight from 66 to 94% [16]. This study suggests the need to pursue quality improvement efforts to promote consistent collection for complete well-child care. These quality improvement efforts can be targeted to particular community-based practices that may be more likely to not consistently document growth measures in the EMR.

### Limitations

This study is based on children who visited a provider participating in MaPCReN, therefore it does not capture all children in Manitoba. Queenan et al. reported that although providers participating in CPCSSN were not representative of all primary care clinicians in Canada, when the patient population is adjusted for age and sex it is representative of the Canadian patient population [24]. This study did not assess the availability of an interdisciplinary team, the provider’s knowledge of current growth measurement recommendations, or if the provider discussed the child’s growth during the appointment. Additionally, we did not have data on all of the patient factors that may have contributed, such as ethnicity. The use of structured EMR data does not include growth measurements obtained but recorded in an encounter note instead of within the EMR exam field. It should be noted that improper recording of measurements in the EMR would be of limited clinical value as they would not automatically populate a growth chart (i.e. WHO percentile curve) and be difficult to review at future visits [31]. Additionally de-identification of the EMR records limited patient birth date to month and year, thus the suggested well-child intervals of 1 to 2 weeks and 1 month were combined. The proportion of patients captured during the < 1 month interval was very low. These patients might have had an inpatient appointment and therefore did not visits a community-based clinic until the child was 1–3 months of age.

## Conclusion

Routine growth monitoring is widely accepted as a standard of well-child care. Pediatricians, urban practices, smaller practices and salaried providers all demonstrated significantly better capture of fully complete growth measures in children. Further studies to explore and understand the impact of growth measures for child health surveillance and elucidation of the developmental origins of disease are possible using this type of data set.

## Data Availability

The datasets generated and/or analysed during the current study are not publicly available due to the confidential nature of the data governed by PHIA legislation, but are available from the corresponding author on reasonable request.

## Abbreviations

EMR: Electronic Medical Record
MaPCReN: Manitoba Primary Care Research Network
CPCSSN: Canadian Primary Care Sentinel Surveillance Network
GEE: Generalized Estimate Eq.
OR: Odds ratios
CI: 95% confidence intervals
SD: Standard Deviation
WHO: World Health Organization
